# Effects of Dual Tasking on Intersegmental Coordination During Walking in People with Parkinson’s Disease: A Cross-Sectional Case–Control Study

**DOI:** 10.3390/geriatrics11030053

**Published:** 2026-04-28

**Authors:** Valéria Feijó Martins, Edilson Fernando de Borba, Lucas de Liz Alves, Leonardo A. Peyré-Tartaruga, Flávia Gomes Martinez

**Affiliations:** 1 Center of Reference for Aging and Movement, Federal University of Rio Grande do Sul, Porto Alegre 90690-200, RS, Brazil; valeria.feijomartins@gmail.com (V.F.M.); fla.gmartinez@gmail.com (F.G.M.); 2 Physical Education Department, Federal University of Paraná, Curitiba 80060-000, PR, Brazil; borba.edi@gmail.com; 3 Department of Public Health, Experimental and Forensic Medicine, University of Pavia, 27100 Pavia, Italy; leonardoalexandre.peyretartaruga@unipv.it

**Keywords:** continuous relative phase, dynamical system, gait, kinematics, locomotion, multitasking behavior

## Abstract

**Background:** In dual-task (DT) conditions, individuals must walk while simultaneously engaging in cognitive or motor tasks, which impacts gait performance, especially in older adults and individuals with Parkinson’s disease (PD). Gait impairments in PD under DT conditions have implications for intersegmental coordination. **Research question:** Intersegmental coordination and gait biomechanics during the DTs were compared between people with PD and older adults. **Methods:** Thirty-two individuals (16 PD, H&Y 1–3; and 16 older adults) participated in this study and were asked to walk under the following self-selected conditions: single task, DT with a math component, and texting on a cell phone. Spatiotemporal, angular, and intersegmental coordination data were collected using a markerless motion analysis system (OpenCap). **Results:** Dual-task conditions significantly affected spatiotemporal and kinematic variables, as well as intersegmental coordination. A significant task effect was observed for thigh–shank coordination, whereas no significant group effect was found for the main coordination outcomes. **Significance:** Significant task effects were observed for intersegmental coordination (thigh–shank CRP), with no significant group differences. The concurrent demands of processing visual and motor information for texting and walking lead to significant reductions in gait speed and lower limb movement, as well as altered intersegmental coordination, with task demands rather than disease status being the primary driver of coordination changes.

## 1. Introduction

Parkinson’s disease (PD) exhibits gait disturbances, a typical motor symptom, even in its earliest stages. Cardinal gait alterations include decreased walking speed, reduced stride length [1,2,3], and compromised postural control, which impact gait variability, asymmetry, and intersegmental coordination [4]. Furthermore, people with PD walk with reduced hip and knee joint excursion, which may further limit their ability to adapt gait strategies at different speeds [5]. In particular, the shorter excursion during the stance phase seems to be related to an impaired pendulum-like mechanism [6,7]. In addition to typical symptoms, cognitive impairment, related to deficits in executive functions, attention, memory, language, and visuospatial aspects, may be six times more prevalent in people with PD compared to the healthy population [8,9]. This affects the performance of tasks that require simultaneous execution of cognitive functions and motor activities, such as walking and talking, or walking while using a cell phone, resulting in difficulties in performing dual-task activities (DT) [4,10].

Larger biomechanical alterations due to dual tasking have been observed in individuals with PD compared to control groups [11,12]. Additionally, gait impairments in PD under DT conditions have implications for functional mobility, as individuals often need to walk while simultaneously engaging in cognitive or motor tasks [13]. It is particularly important that these alterations have been studied using day-to-day practical situations, such as texting on a cell phone while walking. These deficits contribute to increased disability, higher risk of falls, and reduced quality of life among people with PD.

While gait biomechanical alterations are well established in people with PD and older adults, less is known about how DT conditions affect intersegmental coordination through walking. Intersegmental coordination provides insight into the dynamic synergy between joints and may reveal subtle deficits not detectable through traditional spatiotemporal analysis. Comparing the biomechanical and coordination responses of people with PD and age- and sex-matched older adults may help disentangle the contributions of aging and disease-specific impairments. Previous research has shown that dual-task interference in PD is associated with alterations in functional mobility and lower limb strength [10], and that PD-related interference may occur during specific phases of gait, such as late swing and weight transfer [14]. However, most studies have focused on spatiotemporal or joint-level variables, with limited investigation of coordination patterns assessed through continuous relative phase (CRP).

Since dual-task walking depends on executive and visuospatial processing, cognitive status may further influence coordination strategies, particularly under high attentional demand. Therefore, the purpose of this study was to analyze the gait biomechanics and intersegmental coordination between different cognitive tasks during walking. In particular, we aimed to compare the spatiotemporal, joint kinematics, and CRP responses between a widely used dual-task strategy (arithmetic challenge) and a common daily activity (texting on a cell phone) in people with PD and older adults. We hypothesized that the DT condition would result in more impaired intersegmental coordination in the lower limbs of people with PD compared to older adults, due to the impaired brain and neuromuscular resources associated with PD. Secondarily, we hypothesized that intersegmental coordination would be impaired mostly in gait phases more closely related to cortical planning of foot placement [15], as observed previously in gait stability [14].

## 2. Materials and Methods

### 2.1. Study Design 

This cross-sectional case–control study analyzes the baseline data from a clinical trial (NCT06638697). The procedures conformed to the Declaration of Helsinki. Prior to giving consent, all participants were informed about the potential risks and discomforts associated with this study. The Ethics Committee for Research involving Human Beings approved this study (number: 69919017.3.0000.5347).

### 2.2. Participants

A total of 32 subjects participated in this study, comprising 16 people with PD (PD group) and 16 older adults (older group), recruited from the local community project (Reference Center for Aging and Movement, Federal University of Rio Grande do Sul). Recruitment was conducted through community advertisements and newsletters from January to May 2023. The diagnosis of Parkinson’s disease was confirmed by a neurologist according to the Movement Disorder Society (MDS) clinical diagnostic criteria. All participants with PD were in Hoehn and Yahr stages 1 to 3 and had a disease duration of at least two years since diagnosis, confirmed during clinical interview.

All participants with PD were evaluated during the “ON” medication state, approximately 1–2 h after their usual dopaminergic medication intake. The presence of freezing of gait (FOG) and motor fluctuations was screened through a structured clinical interview. No participant reported disabling motor fluctuations that would compromise safe walking performance. An a priori power analysis was conducted using G*Power 3.1 for a repeated-measures ANOVA (between factors). Considering a medium effect size (f = 0.40), α = 0.05, power (1 − β) = 0.80, two groups and three task conditions, the estimated required total sample size was 34 participants; however, 32 were included.

### 2.3. Clinical Assessment

The evaluations were conducted at the Biodynamics Laboratory (LaBiodin) of the Federal University of Rio Grande do Sul. The evaluation session included: a clinical anamnesis to record the main personal data of participants, such as age, gender, education level, housing situation, income, history of falls (history of falls was defined as at least one fall in the previous 12 months, based on self-report during the clinical interview), medication use, medical history, body weight, height, and cognitive function using the Montreal Cognitive Assessment (MoCA) used as a global cognitive screening tool. No exclusion cutoff was applied for MoCA scores; however, participants were required to be able to understand task instructions and cognitive function using the Montreal Cognitive Assessment (MoCA) [16], used as a global cognitive screening tool; application of the H&Y scale to classify PD symptoms [17,18]; an anthropometric evaluation recorded body mass, height and length of the lower limbs.

### 2.4. Experimental Procedure

**Figure 1:**
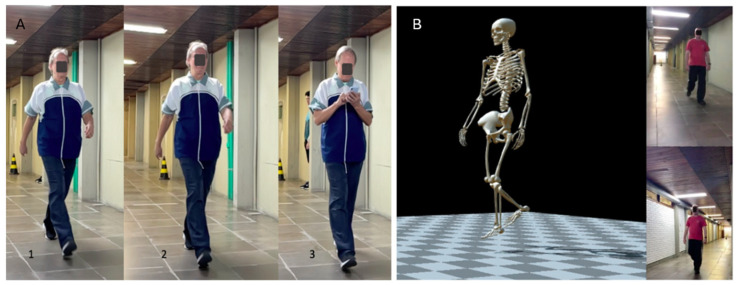
(**A**): Conditions proposed in the test: 1. single task, 2. arithmetic DT, 3. cell phone DT; (**B**): example of scanning by OpenCap.

Following the clinical assessment, participants underwent the biomechanical gait evaluation under the experimental conditions described below. The individuals performed three 10 m walking tests at self-selected speeds in randomized order as follows: (1) the baseline condition where the participants were instructed to walk at their self-selected speed under standard conditions (single task); (2) the motor-cognitive DT involving arithmetic problem-solving (arithmetic DT), participants performed arithmetic calculations aloud using the three times table while walking, for example, they would say “Three times one is three, three times two is six”, and so on; (3) the motor-cognitive DT of composing a message on a cell phone (cell phone task), participants typed the message “I am participating in a walking test at the Aquatic Center” on their cell phones while walking (Figure 1). The walking tests are described in detail elsewhere [19]. The intervals between tests were at least 30 s.

The kinematic records of walking were performed using the OpenCap software (version 1.2.6) [20,21]. For recording, two mobile devices were positioned at a 45-degree angle to the calibrator, which was positioned 7 m from the start of the test (approximately halfway along the route). All records were made in this same recording range. The sampling frequency was 60 Hz (for other information about data collection, see https://www.opencap.ai, accessed on 7 December 2025).

### 2.5. Data Processing

The reconstruction of movement was performed using a markerless motion analysis system (OpenSim, version 4.4, Stanford, CA, USA). This system was used to process segment and joint positions during walking. The analysis of motion data focused on the dominant (right) side, as no significant differences were found between the two sides. We used a customized algorithm in Python 3.10.13 to calculate the spatiotemporal, angular, horizontal velocity, and intersegmental coordination, as outlined in Table 1. A third-order Butterworth low-pass filter with a cutoff frequency of 6 Hz was applied to the signal.

### 2.6. Statistical Analysis

A Shapiro–Wilk test was conducted to assess the normality of the data. Descriptive statistics were calculated for all variables. Between-group comparisons of continuous variables were performed using independent *t*-tests, while categorical variables were analyzed using Poisson regression models. A two-way repeated-measures ANOVA was conducted to examine the main effects of group (PD vs. Older adults), task (single, arithmetic, cell phone), and their interaction. Multiple comparisons between tasks were performed within the group using the post hoc Tukey tests. An effect size analysis (Cohen’s) was applied using the following classification: small 0.20–0.49, medium 0.50–0.79, and large ≥0.80 [28]. The significance level adopted was α = 0.05. All analyses were performed using the statistical software Jasp (Version 0.17.1, The Netherlands).

## 3. Results

The results are organized into sections that focus on non-motor, biomechanics (spatiotemporal and joint kinematics), and intersegmental coordination parameters.

### 3.1. Non-Motor Parameters

The participants’ characteristics are presented in Table 2. No differences were found between the groups for the main sample characteristics, such as sex, education, housing, income, age, and body mass index. The number of medications, diseases, and the visuospatial domain of the MoCA showed significant differences between the groups, with the people with PD group having more diseases (*p* = 0.009), using approximately six medications daily (*p* = 0.002). The PD group had a lower MoCA score in the visuospatial domain (2.6 points) compared to the older group (4.0 points, *p* = 0.013).

### 3.2. Biomechanical and Coordination Parameters

The main and interaction effects are presented in Table 3. Significant task effects were observed for gait speed, stride time, contact phase, and double support time (*p* < 0.050). Significant group effects were found for stride time and selected hip kinematic variables (minimum angle and range of motion), as well as for ankle range of motion. Regarding intersegmental coordination, a significant task effect was observed for thigh–shank CRP (*p* < 0.001), whereas no significant group effect was found (*p* = 0.297), and the interaction effect reached marginal significance (*p* = 0.050). A significant group effect was also found for ankle maximum angle (*p* < 0.001), with the PD group showing reduced ankle extension compared to older adults.

**Figure 2:**
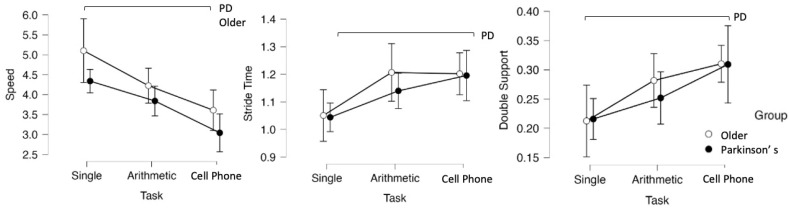
Average and standard deviation values of horizontal speed, stride time, and double support time between the different walking tasks for older adults (white circles) and individuals with Parkinson’s disease (black circles). Horizontal black lines represent statistical differences between tasks.

Gait speed of both groups demonstrated a significant reduction during the cell phone task compared to the single-task condition (*p* < 0.001 for both groups; Figure 2). In the PD group, the stride time was significantly longer during the cell phone task compared to the single-task condition (*p* = 0.040). Additionally, differences were observed in double support time across tasks (*p* = 0.050).

**Figure 3:**
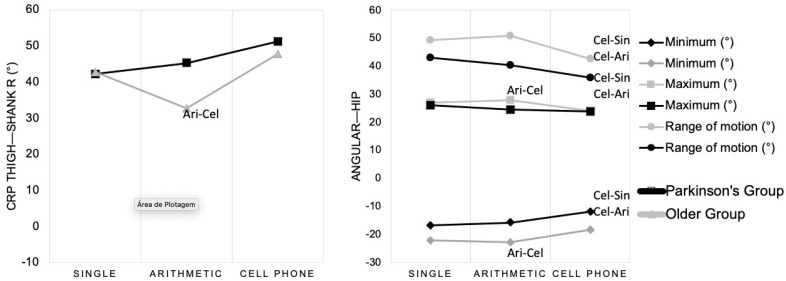
Results of angular and coordinative variables with significant differences between the different walking tasks. Code combinations represent the significant differences (Cel—cell phone; Ari—arithmetic; and Sin—single condition).

Regarding the angular variables of the hip joint, significant differences were observed across tasks, particularly in the PD group (Figure 3). For the MIN knee angle, the PD group showed borderline differences (*p* = 0.050), whereas in the older group, a difference was observed between the arithmetic and cell phone tasks (*p* = 0.012). For the ROM, both groups exhibited significant differences between the cell phone task and the other two conditions. In the PD group, the ROM differed significantly between the single-task and cell phone tasks (*p* < 0.001), and between the arithmetic and cell phone tasks (*p* = 0.042). Similarly, in the older group, significant differences were found between the single-task and cell phone tasks (*p* = 0.002) and between the arithmetic and cell phone tasks (*p* < 0.001). Concerning the MAX hip angle, a significant difference was found only in the older group (*p* = 0.050), with reduced ROM in the cell phone task compared to the arithmetic task condition. These findings suggest that dual-task conditions may influence joint kinematics, particularly in individuals with PD (Figure 3).

Significant differences were observed in the CRP between tasks for specific joint segments. As shown in Table 3 and Figure 3, a significant task effect was found for the right thigh–shank CRP (*p* < 0.001), with a medium effect size. When comparing tasks, the older group exhibited a higher CRP during the cell phone task compared to the arithmetic task (*p* = 0.034), indicating altered intersegmental coordination under dual-task conditions.

Regarding the subphases of the gait cycle, in Table 4, significant differences in CRP were observed in the thigh–shank segments. For the thigh–shank, a significant difference was found in the push-off phase for the PD group between the single-task and cell phone task (*p* = 0.035), suggesting impaired coordination under dual-task conditions. A significant effect of task was observed for thigh–shank CRP, whereas no significant group effect was found, suggesting that dual-task demands, rather than disease status alone, influenced intersegmental coordination. Regarding CRP variability, a significant difference was found between older adults and the PD group during the cell phone condition in the mid-stance phase of the shank–foot segment (OldCel-PDCel), suggesting greater variability in the older group under this condition.

## 4. Discussion

This study aimed to investigate spatiotemporal, joint kinematics and intersegmental coordination during different cognitive tasks during walking in individuals with PD and older adults. Our findings confirmed our main hypothesis, revealing that dual-task conditions altered intersegmental coordination patterns. However, no significant group differences were observed for the main coordination outcomes, suggesting that task demands, rather than disease status alone, primarily influenced intersegmental coordination. Our second hypothesis was rejected due to, in general, unchanged intersegmental coordination between groups and conditions at the different phases of gait. These findings are supported by unchanged gait speed and spatiotemporal parameters between groups, which align with previous results in trained older adults and individuals with PD [5,19]. On the other hand, the speed and spatiotemporal parameters were reduced during the dual task compared to the single task in both groups.

The angular kinematic analysis revealed task and group effects primarily in the hip and knee joints. In the PD group, the cell phone task resulted in reduced knee flexion and range of hip motion compared to both single-task and arithmetic task conditions. Older adults also showed differences between the arithmetic and cell phone tasks, but these were less marked. These findings align with previous research that has demonstrated altered joint kinematics in people with PD [2,3,27]. The reduced hip excursion impacts the stride length in individuals with PD [2]. The reduced knee motion has been related as an important factor underlying the impaired pendulum-like mechanism of gait in PD [6,7].

Our results on intersegmental coordination further support this pattern. While previous findings reported group effects for intersegmental coordination [29], we also observed significant effects of task here, particularly in the thigh–shank CRP, which increased under the cell phone condition in both groups. Notably, changes were observed across both groups, suggesting that increased cognitive load affects coordination patterns regardless of disease status. This may reflect a compensatory adjustment strategy in older adults, whereas individuals with PD may present a more rigid coordination pattern with reduced adaptability under DT demands. Additionally, differences were observed in CRP subphases: the PD group showed altered coordination in the push-off phase of the thigh–shank segment. Previous evidence has shown high activity in the lateral premotor area of the cortex during walking, suggesting that this area is involved in maintaining steady-state gait stability [15]. These findings reinforce the notion that dual tasking can impact temporal intersegmental coordination, particularly under higher cognitive loads [4,23,29].

Considering the known difficulties that individuals with PD face in performing dual tasks (DTs) [4,10], it was expected that the cell phone task—the most complex—would generate the greatest disruptions. Our data support this hypothesis, showing significant gait changes in PD during this task, consistent with Johansson et al. [8], who reported DT costs across multiple gait domains. The cognitive load and visual deprivation imposed by texting while walking likely exacerbates motor control challenges, suggesting that individuals with PD may be more susceptible to attentional interference during walking [13,29,30].

Previous studies with younger populations demonstrated that cell phone use while walking leads to reductions in speed and cadence [13], an increase in step time, and a decrease in mediolateral acceleration, confirming that texting creates a significantly greater interference effect on walking than talking on a cell phone. Our results extend this knowledge to older adults and individuals with Parkinson’s disease, who are more vulnerable to the interference of dual tasking and the risks associated with accidents and falls in everyday situations. This emphasizes the clinical importance of assessing dual-task gait performance in these populations and highlights the potential risks associated with engaging in everyday multitasking while walking. Given the established association between gait disturbances and future falls in older adults, the observed dual-task-related coordination impairments may represent potential markers of fall risk.

Gait impairments and DT costs are strongly associated with increased fall risk in older adults. Prospective population-based studies have demonstrated that gait disorders significantly predict recurrent falls over long-term follow-up, highlighting the importance of rigorous gait assessment in clinical populations [31].

Biomechanical factors, such as decreased hip flexion torque and increased stiffness in the hip and ankle, may contribute to the coordination deficits observed in PD [13]. These adaptations could represent compensatory strategies to maintain balance and stability under dual-task conditions, albeit at the cost of joint mobility and coordination. Studies on gait in other neurological populations, such as multiple sclerosis, support the idea that neurodegenerative conditions impair coordination and motor function, with the complexity of the task further amplifying these deficits [32]. While multiple sclerosis and PD differ in their motor manifestations, both conditions show reduced gait adaptability under cognitive stressors, reinforcing the need for task-specific gait evaluation.

A study on the relationship between cognitive motor capacity and gait in individuals with PD highlights the importance of evaluating the impact of cognitive tasks on walking [26]. Our findings reinforce this necessity, as we observed alterations in both intra- and inter-limb coordination across single- and dual-task conditions. A decline in cognitive performance during dual-task walking, as noted in previous research [33], aligns with our observation of disrupted gait coordination. This suggests a complex interplay between cognitive and motor systems, in which increased cognitive demand can impair not only executive function but also motor coordination in people with PD.

Moreover, the observed differences in MoCA scores between groups may reflect variations in visuospatial processing and executive functioning—both of which are essential for coordinated gait during cognitively demanding tasks [33]. Although the MoCA provides a global cognitive screening measure, it does not allow for a detailed assessment of specific executive or attentional domains that may differentially influence dual-task performance. Future studies should incorporate more comprehensive neuropsychological testing to better understand how domain-specific cognitive impairments interact with intersegmental coordination during DT walking. Therefore, our study provides important evidence for understanding this relationship in PD and highlights the need for interventions that target both cognitive and motor functions to enhance overall performance in daily activities.

To our knowledge, this is the first study to evaluate gait and dual-task conditions in older adults and individuals with PD using OpenCap. The use of a markerless motion analysis system such as OpenCap allowed for detailed biomechanical assessment under ecologically relevant task conditions [20,34,35]. OpenCap enabled the detection of subtle motor adjustments, providing a significant advantage for research in movement disorders and supporting the development of personalized therapeutic strategies. The use of this innovative technology enhances clinical research capabilities by providing objective data and facilitating personalized intervention planning through accurate movement analysis.

Nonetheless, several limitations must be acknowledged. Although all participants with PD had a disease duration of at least two years, we did not quantify disease duration in years, which may limit the ability to interpret the progression-related effects on coordination patterns. The heterogeneity of participants is a potential source of variability; however, we applied matching criteria for age, sex, and other relevant variables to minimize this bias. Additionally, freezing of gait and motor fluctuations were screened through clinical interview but were not formally quantified using validated instruments. Subclinical freezing or fluctuation severity may have influenced gait performance under dual-task conditions. The sample size (n = 32) fell slightly below the a priori estimated requirement of 34 participants, which may have limited the detection of subtle interaction effects, particularly for medium-sized effects. While our results offer valuable insights into coordination deficits in PD, caution is warranted when generalizing findings to broader populations. Additionally, data collection was conducted in a controlled setting, which may not fully replicate real-world walking contexts, potentially affecting ecological validity.

## 5. Conclusions

This study demonstrated that walking while texting on a cell phone significantly altered angular kinematics and intersegmental coordination in individuals with Parkinson’s disease (PD), more so than in cognitively unimpaired older adults. People with PD exhibited distinct coordination patterns, particularly in specific gait subphases such as the thigh–shank, suggesting impaired motor control under increased cognitive load. These findings underscore the vulnerability of gait coordination in PD during complex dual-task situations, highlighting how cognitive demands can exacerbate motor impairments. Therefore, this study reinforces the importance of assessing gait under ecologically valid dual-task conditions and supports the development of individualized interventions aimed at enhancing dual-task performance and functional mobility in individuals with PD.

## Figures and Tables

**Table 1:** Overview of variables processed in OpenSim software, version 4.4.

Variable	Description
Spatiotemporal	The spatiotemporal variables include horizontal velocity, step swing time, contact time, stride length, and stride frequency. The horizontal velocity was calculated to determine functional capacity during walking. Step swing time measures the duration the foot is in the air, while contact time assesses the duration of the foot’s contact with the ground during each step. Stride length measures the anteroposterior distance between two consecutive contacts of the same foot. Stride frequency indicates the number of strides per second [22].
Joint kinematics	The joint kinematics include the minimum angle (Min), the maximum angle (Max) and the range of motion (ROM). The minimum angle represents the smallest angle recorded, representing the most flexed position of the joint, while the maximum angle corresponds to the largest angle recorded during a stride cycle, representing the most extended position of the joint. The range of motion is the difference between the maximum angle and the minimum angle, reflecting the joint’s ability to move [23].
Intersegmental coordination—continuous relative phase (CRP)	The body’s intersegmental coordination of the shank–foot and thigh–shank at sagittal plane, and shoulder–pelvis at transversal plane couplings were quantified using average continuous relative phase (CRP) [24]. A CRP of 0° signifies in-phase movement, while non-zero CRP indicates out-of-phase movement. A CRP of 180° denotes anti-phase coupling. Positive CRP values indicate the proximal segment (thigh) leads, while negative values indicate the distal segment (shank) leads [23,25,26]. We also derived phase portraits using position/velocity phase planes.
CRP variability	The intersegmental coordination stability, here called CRP variability, was quantified using the standard deviation of CRP on the same segment couplings [27]. The CRP variability denotes the stability of the local motor system. Higher values of CRP variability mean major likelihood to adjust the movement pattern facing unexpected conditions. All data were time-normalized to 100% of the gait cycle before CRP calculation [23,25,26].
CRP subphases	We applied the Hilbert transform due to the method’s robustness to frequency artifacts. The data were normalized and divided into the total contact phase, load response (0–20% of the contact phase), medium support (20–50% of the contact phase), terminal support (50–80% of the contact phase) and thrust (80–100% of the contact phase) in each joint combination (Mehdizadeh & Glazier, 2018) [23]. We also present the CRP analysis for the right side of each joint combination.

**Table 2:** Participant characteristics.

Variables		Older Group	Parkinson’s Group	*p*
Sex (%)	Female	66.7	40	0.456
	Male	33.3	60	
Schooling (%)	University education	100	85.7	0.715
	High school	-	14.3	
Dwelling (%)	Alone	35.7	23.1	0.802
	Not alone	64.3	76.9	
Income (%)	High income	28.5	16.6	0.820
	Not high income	71.4	83.3	
History of falls (%)	Fall	21.4	30.7	0.829
	Do not fall	78.6	69.3	
Medications		2.6	6.7	**0.002**
Disease		0.8	2.1	**0.009**
Age (years)		70.8 ± 4.7	66.7 ± 8.4	0.110
BMI (kg/m^2^)		26.9 ± 3.1	29.1 ± 6.8	0.302
Body mass (kg)		73.5 ± 12.0	79.3 ± 22.1	0.405
Height (m)		1.65 ± 0.11	1.64 ± 0.09	0.889
MoCA (points)		24.4 ± 3.1	23.4 ± 4.2	0.505
MoCA-visuospatial/executive (points)		4.0 ± 0.9	2.6 ± 1.7	**0.013**
MoCA-naming (points)		2.4 ± 0.6	2.3 ± 1.1	0.691
MoCA-attention (points)		4.8 ± 1.1	4.0 ± 1.9	0.138
MoCA-language (points)		2.1 ± 0.9	1.6 ± 0.9	0.249
MoCA-abstraction (points)		1.8 ± 0.4	1.6 ± 0.7	0.367
MoCA-delayed recall (points)		3.2 ± 1.6	3.1 ± 2.0	0.848
MoCA-orientation (points)		5.9 ± 0.2	5.0 ± 2.1	0.109

Note: body mass index (BMI), Montreal Cognitive Assessment (MoCA). Data are presented as mean ± standard deviation unless otherwise specified. All participants with Parkinson’s disease had a disease duration of at least two years and were evaluated during the ON medication state. Bold indicates statistically significant differences (*p* < 0.05). Percentages were calculated based on available data for each variable.

**Table 3:** Main and interaction factor effects on outcome measures.

Variables	Task	Group	Task × Group
**Spatiotemporal**			
Speed (km/h)	**F(9.838); *p* < 0.001;** **ƞ^2^p = 0.210**	F(0.151); *p* = 0.699; ƞ^2^p = 0.002	F(0.464); *p* = 0.630; ƞ^2^p = 0.010
Stride time (s)	**F(10.060); *p* < 0.001;** **ƞ^2^p = 0.203**	**F(5.026); *p* = 0.028;** **ƞ^2^p = 0.051**	F(0.483); *p* = 0.619; ƞ^2^p = 0.010
Contact phase (%)	**F(3.341); *p* = 0.041;** **ƞ^2^p = 0.082**	F(0.009); *p* = 0.924; ƞ^2^p < 0.001	F(1.035); *p* = 0.360; ƞ^2^p = 0.025
Double support (s)	**F(8.676); *p* < 0.001;** **ƞ^2^p = 0.339**	F(0.091); *p* = 0.765; ƞ^2^p = 0.002	F(0.308); *p* = 0.737; ƞ^2^p = 0.005
**Joint kinematics (degrees)**			
Knee—Minimum	F(3.181); *p* = 0.050; ƞ^2^p = 0.009	F(0.035); *p* = 0.853; ƞ^2^p = 0.001	F(0.368); *p* = 0.694; ƞ^2^p = 0.001
Knee—Maximum	F(0.235); *p* = 0.791; ƞ^2^p = 0.002	F(0.004); *p* = 0.949; ƞ^2^p = 0.005	F(0.029); *p* = 0.971; ƞ^2^p = 0.002
Knee—Range of motion	F(0.610); *p* = 0.548; ƞ^2^p = 0.006	F(0.002); *p* = 0.968; ƞ^2^p = 0.001	F(0.103); *p* = 0.902; ƞ^2^p = 0.001
Hip—Minimum	**F(3.878); *p* = 0.025;** **ƞ^2^p = 0.081**	**F(14.334); *p* < 0.001;** **ƞ^2^p = 0.150**	F(0.115); *p* = 0.891; ƞ^2^p = 0.003
Hip—Maximum	**F(5.622); *p* = 0.007;** **ƞ^2^p = 0.026**	F(0.322); *p* = 0.576; ƞ^2^p = 0.012	F(2.144); *p* = 0.129; ƞ^2^p = 0.010
Hip—Range of motion	**F(25.892); *p*< 0.001;** **ƞ^2^p = 0.128**	**F(8.628); *p* = 0.007;** **ƞ^2^p = 0.203**	F(2.502); *p* = 0.093; ƞ^2^p = 0.012
Ankle—Minimum	F(1.883); *p* = 0.159; ƞ^2^p = 0.048	F(1.374); *p* = 0.245; ƞ^2^p = 0.018	F(0.058); *p* = 0.943; ƞ^2^p = 0.001
Ankle—Maximum	F(1.112); *p* = 0.335; ƞ^2^p = 0.025	**F(13.070); *p* < 0.001;** **ƞ^2^p = 0.146**	F(0.514); *p* = 0.600; ƞ^2^p = 0.012
Ankle—Range of motion	F(1.051); *p* = 0.358; ƞ^2^p = 0.021	**F(4.993); *p* = 0.035;** **ƞ^2^p = 0.090**	F(1.196); *p* = 0.312; ƞ^2^p = 0.023
**Intersegmental coordination (degrees)**			
CRP shank–foot	F(1.145); *p* = 0.327; ƞ^2^p = 0.018	F(1.937); *p* = 0.177; ƞ^2^p = 0.048	F(0.732); *p* = 0.487; ƞ^2^p = 0.011
CRP thigh–shank	**F(8.134); *p* < 0.001;** **ƞ^2^p = 0.087**	F(1.140.); *p* = 0.297; ƞ^2^p = 0.030	F(3.209); *p* = 0.050; ƞ^2^p = 0.034
CRP shoulder–pelvis	F(2.066); *p* = 0.138; ƞ^2^p = 0.013	F(2.922); *p* = 0.101; ƞ^2^p = 0.095	F(0.512); *p* = 0.603; ƞ^2^p = 0.003
**CRP variability**			
DP CRP shank–foot	F(1.249); *p* = 0.296; ƞ^2^p = 0.018	F(1.560); *p* = 0.224; ƞ^2^p = 0.041	F(0.524); *p* = 0.595; ƞ^2^p = 0.008
DP CRP thigh–shank	F(1.919); *p* = 0.158; ƞ^2^p = 0.037	F(0.324); *p* = 0.575; ƞ^2^p = 0.007	F(0.343); *p* = 0.712; ƞ^2^p = 0.007
DP CRP shoulder–pelvis	F(1.282); *p* = 0.287; ƞ^2^p = 0.011	F(0.407); *p* = 0.530; ƞ^2^p = 0.013	F(1.035); *p* = 0.363; ƞ^2^p = 0.009

Note: CRP: continuous relative phase. For each effect, values represent the F-statistic, *p*-value, and partial eta squared (η^2^p). The Task × Group term represents the interaction effect, indicating whether the effect of task differs between groups. Bold values indicate statistically significant effects (*p* < 0.05).

**Table 4:** Average continuous relative phase at different phases of gait (loading response, mid-stance, terminal stance and push-off phases), and at different conditions (Cel—cell phone; Ari—arithmetic; and Sin—single condition) for individuals with Parkinson’s disease and older adults.

Coupled Joints	Gait Cycle Subphase (Degree)	Groups and Tasks						Comparison
		Older Group			Parkinson’s Group			
		Sin	Ari	Cel	Sin	Ari	Cel	
**Shoulder–Pelvis**	**Loading Response**	9.94	10.4	13.8	11.6	11.6	10.1	N/D
	Mid Stance	5.2	6.9	6.5	5.5	5.8	6.5	N/D
	Terminal Stance	10.0	8.3	9.3	8.8	8.2	8.9	N/D
	Push Off	8.1	8.1	8.7	7.1	7.2	7.6	N/D
Shank–Foot	Loading Response	33.7	45.5	46.7	38.1	39.4	26.4	N/D
	Mid Stance	29.3	31.2	31.7	33.0	33.7	28.5	N/D
	Terminal Stance	52.7	34.2	33.3	36.2	25.9	34.8	N/D
	Push Off	40.8	38.2	34.1	27.4	22.7	34.6	N/D
Thigh–Shank	Loading Response	37.2	45.0	37.9	62.5	58.2	46.3	N/D
	Mid Stance	38.6	43.5	45.7	52.7	52.5	48.5	N/D
	Terminal Stance	44.3	42.6	49.6	46.7	47.5	56.7	N/D
	Push Off	50.9	39.2	55.4	35.4	43.8	62.1	PDSin-PDCel
Variability								
Shoulder–Pelvis	Loading Response	1.7	1.5	1.8	1.5	1.5	1.5	N/D
	Mid Stance	4.2	4.6	5.0	4.8	4.6	4.5	N/D
	Terminal Stance	2.8	2.3	2.8	2.3	2.5	2.6	N/D
	Push Off	9.2	7.7	8.3	7.7	7.6	7.5	N/D
Shank–Foot	Loading Response	9.2	11.8	18.0	18.2	14.4	7.2	N/D
	Mid Stance	14.5	13.1	18.5	10.9	11.0	8.1	OldCel-PDCel
	Terminal Stance	34.3	20.7	13.7	14.6	7.5	15.1	N/D
	Push Off	10.6	7.8	7.9	7.8	6.6	10.2	N/D
Thigh–Shank	Loading Response	6.4	7.6	6.5	4.7	8.9	6.8	N/D
	Mid Stance	6.5	5.3	8.0	8.2	6.9	8.1	N/D
	Terminal Stance	9.9	9.6	10.4	8.8	12.2	9.6	N/D
	Push Off	3.1	4.5	4.9	3.4	4.6	4.9	Sin-Cel

## Data Availability

The data presented in this study are available on request from the corresponding author. The data are not publicly available due to restrictions related to participant privacy.
